# Hierarchical Micro-/Nano-Structures on Polycarbonate via UV Pulsed Laser Processing

**DOI:** 10.3390/nano10061184

**Published:** 2020-06-17

**Authors:** Marek Mezera, Sabri Alamri, Ward A.P.M. Hendriks, Andreas Hertwig, Anna Maria Elert, Jörn Bonse, Tim Kunze, Andrés Fabián Lasagni, Gert-willem R.B.E. Römer

**Affiliations:** 1 Department of Mechanics of Solids, Surfaces and Systems (MS3), Faculty of Engineering Technology, University of Twente, Drienerlolaan 5, 7522 NB Enschede, The Netherlands; g.r.b.e.romer@utwente.nl; 2 Fraunhofer Institut für Werkstoff- und Strahltechnik (IWS), Winterbergstraße 28, 01277 Dresden, Germany; sabri.alamri@iws.fraunhofer.de (S.A.); tim.kunze@iws.fraunhofer.de (T.K.); andres_fabian.lasagni@iws.fraunhofer.de (A.F.L.); 3 Optical Science Group, MESA + Institute for Nanotechnology, University of Twente, Drienerlolaan 5, 7500 AE Enschede, The Netherlands; w.a.p.m.hendriks@utwente.nl; 4 Bundesanstalt für Materialforschung und -prüfung (BAM), Unter den Eichen 87, 12205 Berlin, Germany; andreas.hertwig@bam.de (A.H.); anna-maria.elert@bam.de (A.M.E.); joern.bonse@bam.de (J.B.); 5 Institut für Fertigungstechnik, Technische Universität Dresden, Georg-Bähr-Str. 3c, 01069 Dresden, Germany

**Keywords:** Direct Laser Interference Patterning, Laser-induced Periodic Surface Structures, polycarbonate, hierarchical structures, surface functionalization

## Abstract

Hierarchical micro/-nanostructures were produced on polycarbonate polymer surfaces by employing a two-step UV-laser processing strategy based on the combination of Direct Laser Interference Patterning (DLIP) of gratings and pillars on the microscale (3 ns, 266 nm, 2 kHz) and subsequently superimposing Laser-induced Periodic Surface Structures (LIPSS; 7–10 ps, 350 nm, 100 kHz) which adds nanoscale surface features. Particular emphasis was laid on the influence of the direction of the laser beam polarization on the morphology of resulting hierarchical surfaces. Scanning electron and atomic force microscopy methods were used for the characterization of the hybrid surface structures. Finite-difference time-domain (FDTD) calculations of the laser intensity distribution on the DLIP structures allowed to address the specific polarization dependence of the LIPSS formation observed in the second processing step. Complementary chemical analyzes by micro-Raman spectroscopy and attenuated total reflection Fourier-transform infrared spectroscopy provided in-depth information on the chemical and structural material modifications and material degradation imposed by the laser processing. It was found that when the linear laser polarization was set perpendicular to the DLIP ridges, LIPSS could be formed on top of various DLIP structures. FDTD calculations showed enhanced optical intensity at the topographic maxima, which can explain the dependency of the morphology of LIPSS on the polarization with respect to the orientation of the DLIP structures. It was also found that the degradation of the polymer was enhanced for increasing accumulated fluence levels.

## 1. Introduction

In the course of evolution, flora and fauna adapted distinct surface structures, which induced specific functionalities and therefore ensured survival and procreation. A well-known example of a functional surface found in nature is the lotus leaf, which is water repellent and self-cleaning [1]. Other examples are the wings of butterflies and cicada, which are bactericidal [2,3,4], or the skin of sharks, which present self-cleaning, anti-biofouling, hydrodynamic and drag reduction properties [5]. Other examples, like the tenebrionid beetle *Stenocara* collects drinking water on its integument from morning fog and transports the collected water on its skin towards its mouthparts [6]. In this way, the insect can survive to the Namibian desert climate. Similar functionalities can be also found on moisture harvesting lizards [7]. All of these different specific surface properties are the result of so-called functionalized surfaces, which often consist of regular (hierarchical) micro- and nanometer sized surface structures. Functionalized surfaces have received increased scientific attention in recent years, aiming to reproduce them (biomimetics) due to their potential for new applications, such as anti-bacterial hip implants [8,9], increased [10] or decreased [11] cell-tissue growth onto implantable materials, liquid motion flow in microfluidics [12], fluid transport in tribological systems [13], friction control [14,15], wettability control [9,16,17] or colorization of surfaces [18,19,20,21].

Two well established laser-based methods have been shown in the past to be capable of creating micro- and nanostructured surfaces directly on the materials surface and thus functionalizing them, namely *Direct Laser Interference Patterning* (DLIP) and *Laser-induced Periodic Surface Structures* (LIPSS). DLIP is a method that produces micrometer and sub-micrometer sized, regular (hierarchical) structures on various materials, such as metals [17], polymers [16,22] or ceramics [23]. The periodic structures are created due to the interference pattern, which is produced when two or more laser beams are overlapped, leading to material removal (ablation) at the interference maxima of the spatially modulated intensity distribution. In the case of two-beam interference, the spatial period of the interference pattern can be controlled by the laser wavelength (λ) and the angle of incidence of the interfering laser beams (θ) [17]. Employing modern laser and beam scanning technology, the DLIP technique can fulfill industrial demands by addressing individual patterns of several square micrometer areas only, at processing rates of 0.9 $m^{2}/\min$ and 0.3 $m^{2}/\min$ for polymers and metals, respectively [24].

The second approach is based on LIPSS. LIPSS are regular (hierarchical) micro- to nanometer sized surface ripples, which appear due to the interference of (1) the impinging laser radiation with its scattered light at the surface or (2) laser triggered surface plasmon polaritons [25,26,27] and can be processed on solids due to polarized, (ultra-) short pulsed laser irradiation at laser peak fluence levels close to the ablation threshold [25,28]. The direction of the LIPSS depends on the material and the (linear) laser beam polarization. Their periodicity depends on several process parameters, such as the laser wavelength (λ), the angle of incidence (α), the number of pulses processing effectively impinging one spot ($N_{\mathrm{eff}}$) and the laser peak fluence ($F_{0}$) [9,25,28]. Several types of LIPSS can be distinguished, depending on the laser processing parameters and the material, e.g., common *Low Spatial Frequency LIPSS* (LSFL) with a period of about the laser wavelength ($\Lambda_{\mathrm{LIPSS}}∼\lambda$), *High Spatial Frequency LIPSS* (HSFL) with a period well below the laser wavelength ($\Lambda_{\mathrm{LIPSS}}<\lambda /2$), or even *hexagonally arranged triangular nanopillars* with an overall period close to the laser wavelength ($\Lambda_{\mathrm{LIPSS}}∼\lambda$) [25,28]. LIPSS can be processed on metals [8,9,10,18,19,20,29], semiconductors [12,21,25,28], dielectrics [30], ceramics [31,32,33] and polymers [34,35,36,37]. Large area processing of LIPSS is easily achieved in a one-step approach by scanning the focused laser beam in a meandering way across the sample surface. Since the central high fluence part of the Gaussian laser beam profile can generate different types of LIPSS than its low fluence wing, hierarchical micro-/nano-structures are easily feasible [9,38]. Moreover, as the periodicity of HSFL is not constrained by the optical diffraction limit, these extremely fine nanostructures may be even superimposed to sub-micrometric LSFL [39].

While individual scientific communities have already independently explored and optimized the processing of LIPSS and DLIP-based structures in detail [25,40,41], the combination of both techniques is still widely unexplored [42]. Although hierarchical structures can be achieved by using LIPSS [9,38] or DLIP [16] methods separately, the hybrid two-step laser process can provide an enhanced flexibility control of the surface features as well as explore new geometries in view of tailored surface functionalities.

Commercially available polycarbonate is used as sample material due to its wide range of applications, such as for products in the electronic and the automotive sector, in building and construction and for optical information storage systems, because of its unique combination of properties such as excellent toughness, high electrical insulation, transparency and large heat distortion resistance [43]. However, UV radiation leads to depolymerization of the molecular structure of polycarbonate [44,45,46,47] and this material modification could impair the use for applications. Hence, the quantitative changes of the molecular structure due to the two laser based surface functionalization techniques need to be assessed.

In this work, the evolution of nanometer sized LSFL on top of different types of micrometer sized DLIP structures on polycarbonate with respect to the laser peak fluence is studied, depending on the morphology and dimensions of the DLIP structures, as well as the direction of the laser polarization relative to the orientation of the DLIP structure, to obtain hierarchical structures. Additionally, the structural molecular changes of the polycarbonate due to the laser irradiation are studied.

## 2. Materials and Methods

Commercially available Bisphenol-A polycarbonate (PC) plates (Makrolon™ of Covestro AG, Leverkusen, Germany) with a thickness of 5 mm and a surface roughness of $R_{a}\approx 2$ nm were used as samples. DLIP and LIPSS methods were used on the samples using three different laser setups; one at the University of Twente (TruMicro 5050 of Trumpf GmbH, Ditzingen, Germany) and two at the Faunhofer Institute for Material and Beam Technology IWS (DLIP-μFab, Fraunhofer IWS, Dresden, Germany; Fuego of Time-Bandwidth Products AG, Zurich, Switzerland), as listed in Table 1.

### 2.1. Direct Laser Interference Patterning Configurations

The structuring of the PC samples was conducted by a compact two-beam DLIP system (DLIP-μFab, Fraunhofer IWS, Dresden, Germany), which produces confined DLIP treated areas containing the periodic structures created per laser pulse (also called pixels), with a diameter of $d_{P}\approx$ 25 μm. The pixel diameter was calculated using the D-squared method described elsewhere [48]. The system uses a frequency quadrupled Q-switched laser head (TECH-263 Advanced of Laser-export Co. Ltd., Moscow, Russia) with a maximum pulse energy of 50 μJ and operating at a wavelength of λ= 263 nm and a pulse duration shorter than 3 ns. The laser beam has a nearly Gaussian intensity distribution (${\mathrm{TEM}}_{00}$) with a beam quality of $M^{2}<1.3$. The setup of the used DLIP optics allows the primary beam from the laser source to split into two single beams by means of a diffractive optical element. The sub-beams are parallelized by a prism and finally overlapped at the sample surface using a focusing aspheric lens.

**Figure 1:**
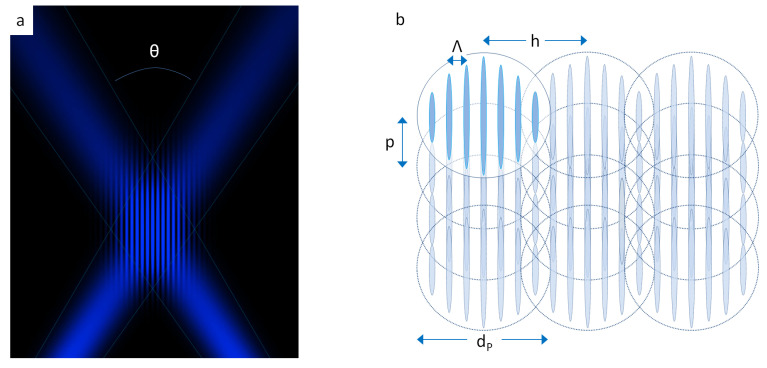
(**a**) Depiction of the interference phenomenon between two laser beams overlapping with an angle θ and (**b**) Scheme of the texturing approach for the displacement of several DLIP pixels on the sample’s surface with *p*: pulse separation; *h*: hatch distance; $d_{p}$: pixel size; Λ: DLIP period. The scanning direction is vertical.

As can be observed in Figure 1a, an interference pattern is obtained within the volume where the two single laser beams overlap. Changing the position of the prism modifies the interfering angle θ, and leads to a change of the spatial period Λ of the periodic structures. In the employed setup, spatial periods Λ in the range between 1.0 μm and 11.0 μm can be produced. In order to structure larger areas than the DLIP pixel, the sample is moved using a high precision computer-controlled stage system (PRO155-05, Aerotech GmbH, Fürth, Germany), resulting in square-shaped processed areas with an edge length of 30 mm full covered with a ridge-like pattern. In particular, the samples were moved in the direction parallel to the interference lines with a spatial pulse separation *p* and successively displaced laterally of a quantity *h* (hatch distance), chosen as an integer of the spatial period (see Figure 1b). Moreover, for producing micro-“pillars”, the areas treated with DLIP and containing a ridge-like pattern have been rotated by 90 degrees and re-irradiated, selectively ablating the previous pattern in the interference maxima. Note, that we use the term “pillar” here in the following for simplicity, since micro-“cones” are usually referred to as self-assembling surface structures [49,50,51].

### 2.2. Fabrication of Laser-Induced Periodic Surface Structures

**Figure 2:**
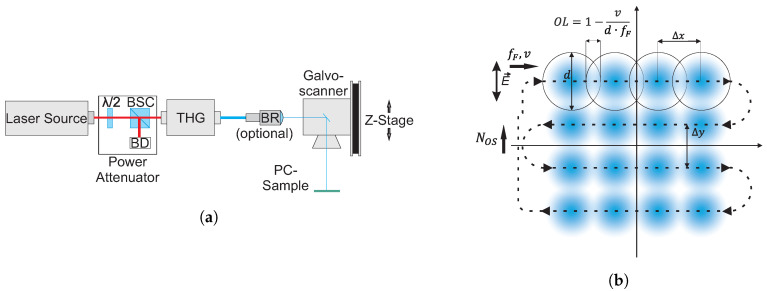
(**a**) Schematic representation of the laser setup; λ/2: half-wave plate; BSC: polarizing beam splitter cube; BD: beam dump; THG: third harmonic generator, BR: beam reducer. (**b**) Scanning trajectory of the laser spot; the double-headed arrow indicates the direction of the laser polarization $\vec{E}$; $f_{F}$: laser pulse frequency; *v*: scan velocity; *d*: laser spot diameter; OL: geometrical pulse-to-pulse overlap; $N_{\mathrm{OS}}$: number of overscans; Δx: geometrical pitch between subsequent laser pulses in *x*-direction, Δy: Line pitch in *y*-direction.

For the manufacturing of LIPSS two laser sources were used, see Table 1. Third harmonics were generated of a pulsed Yb:YAG disk laser source (TruMicro 5050 of Trumpf GmbH, Ditzingen, Germany) emitting a linearly polarized laser beam with a wavelength of 1030 nm, diameter of ≈5 mm, a maximum pulse repetition rate of 400 kHz, pulse energies up to 125 μJ and a fixed pulse duration of 6.7 ps. In other experiments, a frequency-tripled Nd:VAN laser source (Fuego of Time-Bandwidth Products AG, Zurich, Switzerland) emitting a linearly polarized laser beam with a wavelength of 1064 nm, a maximum pulse repetition rate of 8 MHz, pulse energies up to 200 μJ and a fixed pulse duration of 10 ps were used. To obtain homogeneous areas of LIPSS, the laser beam was scanned over the substrate using galvanometer scanners (intelliSCAN14 of ScanLab GmbH, Puchheim, Germany). The laser beam was focused on the surface of the samples, using a telecentric Fθ lens (Ronar of Linos GmbH, Göttingen, Germany) with a focal length of 103 mm. For obtaining large geometrical pulse-to-pulse overlap values in both x− and y−directions at a scan speed of 1 m/s, the laser spot diameter on the sample was increased either by decreasing the laser beam diameter to ≈1 mm before focusing using a beam reducing telescope (TRE13 of Optogama, Vilnius, Lithuania), or by defocused laser processing. It was shown in an earlier publication, that the LIPSS morphologies and dimensions do not significantly differ when processing a defocused laser beam compared to processing with the focal spot [52]. The geometrical pulse-to-pulse overlap is given by $OL=\left(1-v/(d\times f_{F})\right)\times 100$, with *v* being the laser scan speed, *d* the beam spot diameter and $f_{F}$ the laser pulse repetition rate. The processing parameters for the manufacturing of LIPSS are also summarized in Table 1. The meandering area scanning procedure could be repeated several times, denoted as the number of overscans ($N_{\mathrm{OS}}$). Schematic representations of the laser setups and the scanning trajectory of the laser spot are shown in Figure 2.

The laser power at the sample surface was measured using a photodiode power sensor (S130VC of ThorLabs GmbH, Dachau, Germany) with a measurement uncertainty of ±5%, connected to a readout unit (PM100A of ThorLabs GmbH, Dachau, Germany). Along with the pulse repetition rate, this allowed to determine the energy *E* per individual laser pulse. The Gaussian (${\mathrm{TEM}}_{00}$) focal spot diameter d=174±1.6μm ($e^{-2}$) was measured in the sample processing plane using a laser beam characterization device (MicroSpotMonitor of Primes GmbH, Pfungstadt, Germany). From both information, the peak laser fluence $F_{0}$ in front of the sample surface was calculated according to $F_{0}=\frac{2E}{\pi {d/2}^{2}}$ [48].

### 2.3. Morphological Characterization

The morphology and dimensions of the processed surface structures were analyzed by a Scanning Electron Microscope (SEM JSM-7200F of JEOL, Tokio, Japan) and an Atomic Force Microscope (AFM NX10, Park Systems Corp., Suwon, Korea) in true non-contact mode using a non-contact cantilever (PPP-NCHR, $125\;\times 30\;\times \;4\;\mu m^{3}$, nominal tip radius < 10 nm, Park Systems Corp., Suwon, Korea). Prior to SEM characterization, the samples were sputter coated with gold (JFC-1300 coater from JEOL, Tokio, Japan), resulting in a ≈10 nm thick, electrically conductive layer.

From SEM micrographs, the spatial frequencies of LIPSS were analyzed with the help of the 2D fast Fourier transform (FFT) algorithm using a MATLAB script [53]. Details of this script are reported in our earlier work [28]. From cross-sections of AFM micrographs, the amplitude of LIPSS were determined using another MATLAB script, also reported in [52].

### 2.4. FDTD Simulations

**Figure 3:**
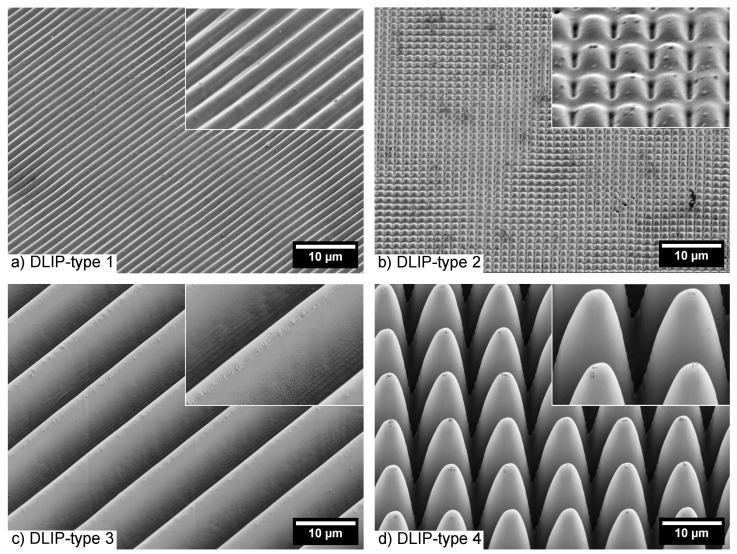
(**a**) ridge-like DLIP structure with a period of 1.5 μm and an depth of 400 nm (DLIP-type 1); (**b**) pillar-like DLIP structure with a period of 1.5 μm and an depth of 400 nm (DLIP-type 2); (**c**) ridge-like DLIP structure with a period of 10 μm and an depth of 15 μm (DLIP-type 3); (**d**) pillar-like DLIP structure with a period of 10 μm and an depth of 15 μm (DLIP-type 4) obtained with laser parameters listed in Table 1.

A commercially available photonic *Finite-difference time-domain* (FDTD) simulation software (Lumerical FDTD of Lumerical Inc., Vancouver, Canada) was used to analyze numerically the time-averaged optical intensity distribution of one laser pulse duration induced by a 6.7 ps laser pulse with a wavelength of 343 nm and with the laser beam polarization perpendicular and parallel to 1.5 μm ridge-like DLIP structures on polycarbonate (DLIP-type 1, see Figure 3a). The surface of the DLIP structure was modeled using the period, depth and full width at half maximum (FWHM) of the DLIP-type 1 structure obtained by AFM measurements. The period was found to be 1.5 μm, the depth of was found to be 400 nm and the FWHM of the ridges was found to be about 1 μm. The mesh settings of the two-dimensional computations were set to an automated mesh accuracy of 7 with a minimal step size of 0.25 nm, the time step size was set to 1.62$\times 10^{-17}$ s, and the periodic boundaries were periodical on *x*-axis were set to *Periodic* and on the *y*-axis to *PML*. The optical properties were taken from Ref. [54].

### 2.5. Chemical Characterization

In order to analyze the surface chemistry of the polycarbonate substrate before and after laser irradiation of the sample, two different spectroscopy techniques were employed to record the IR spectra, i.e., *micro-Raman spectroscopy* (μ-RS) and *microscopy based Fourier-transform infrared spectroscopy* (FTIR).

μ-RS was performed on the laser-irradiated sample and on a reference position (Alpha 300R, WiTEC, Ulm, Germany). A ruled 600 grooves/mm grating was chosen in the optical spectrometer (UHTS 300, WiTEC, Ulm, Germany), which was equipped with a Peltier-cooled CCD camera (iDus DV401A, Andor Technology Ltd, Belfast, Ireland) operated at a temperature of 210 K. The resulting wavenumber resolution is < 2 ${\mathrm{cm}}^{-1}$. The ps-laser irradiated surface regions were excited at a power level of 0.6 mW using the 532 nm emission line of a continuous wave laser (Excelsior, Spectra Physics, Santa Clara, USA). The Raman-laser radiation was focused on the sample surface by a microscope objective (EC Epiplan 20× NA 0.4, Carl Zeiss AG, Oberkochen, Germany) probing a circular spot of about 4 μm in diameter. All spectra are presented without background correction.

FTIR spectra were recorded in attenuated total reflection (ATR) mode (Vertex 70 with a Hyperion 3000 microscope, Bruker Optik, Ettlingen, Germany). The ATR microscope objective is equipped with a Ge-crystal tip ensured surface sensitivity through evanescent field coupling. ATR FT-IR spectra were taken at numerous arbitrary positions on laser processed and unprocessed sample areas with a measurement area of 80×80 $\mu m^{2}$. The FTIR spectra were background corrected (see details below) and absorption peaks resulting from ambient air containing CO${}_{2}$ water vapor were removed.

## 3. Results and Discussion

### 3.1. Direct Laser Interference Patterning on Polycarbonate

Four different types of DLIP structures were processed in a first set of experiments. DLIP-type 1 and 2 are ridge- and pillar-like structures, respectively, with a period of 1.5 μm and a height of approximately 400 nm (Figure 3a,b). Otherwise, DLIP-type 3 and 4 types are ridge- and pillar-like structures, respectively, with a period of 10 μm and an approximated height of about 15 μm (Figure 3c,d). The laser processing parameters are listed in Table 1.

### 3.2. Laser-Induced Periodic Surface Structures on Polycarbonate

**Figure 4:**
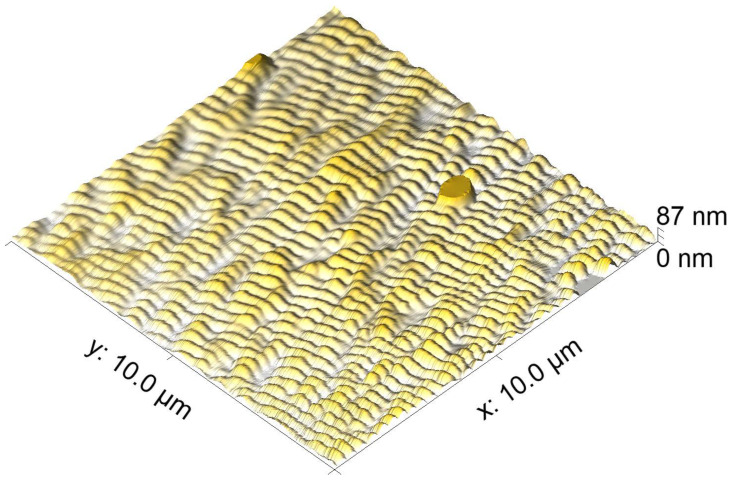
AFM topography of LSFL processed on untreated polycarbonate with the above mentioned parameters.

In a second processing approach, an area of 5 × 5 ${\mathrm{mm}}^{2}$ was processed with the Trumpf TruMicro 5050 laser system on a pristine polycarbonate sample by scanning the laser spot perpendicular to the laser polarization and using an overlap of OL=93% at a pulse frequency of *f* = 100 kHz, number of overscans $N_{\mathrm{OS}}=1000$ and a peak fluence level of $F_{0}$ = 4.42 mJ/cm${}^{2}$. These parameters permitted low-spatial frequency LIPSS parallel to the laser polarization (type II [25]) with a very homogeneous morphology, see Figure 4. The period of the LSFL-II was found to be $\Lambda_{\mathrm{LIPSS}}=265\pm 75$ nm and their amplitude (modulation depth) *A* was 11±8 nm. On the basis of these laser processing parameters, hierarchical micro-/nanostructures were produced by processing LSFL-II on top of the four different types of DLIP structures (see next section).

### 3.3. Hierarchical Structures on Polycarbonate

**Figure 5:**
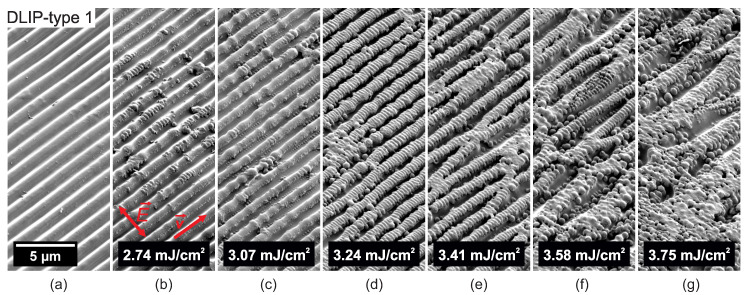
SEM micrographs of (**a**) ridge-like DLIP structure with a period of 1.5 μm (DLIP-type 1). (**b**–**g**) Evolution of surface morphology on top of the DLIP structure upon additional scan-processing with increasing peak fluence levels and a laser beam polarization perpendicular to the DLIP ridges processed with the Trumpf TruMicro 5050 laser system.

**Figure 6:**
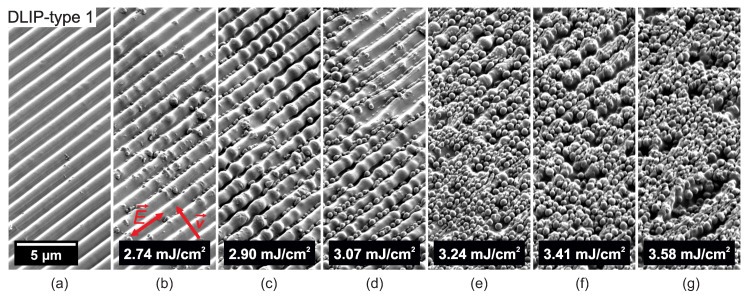
SEM micrographs of (**a**) ridge-like DLIP structure with a period of 1.5 μm (DLIP-type 1). (**b**–**g**) Evolution of surface morphology on top of the DLIP-type 1 structure with increasing peak fluence levels and a laser polarization parallel to the DLIP ridges processed with the Trumpf TruMicro 5050 laser system.

**Figure 7:**
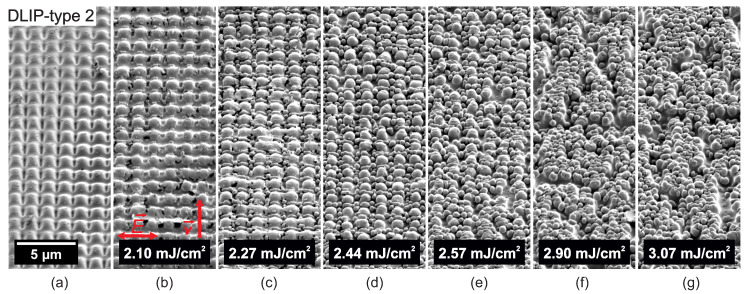
SEM micrographs of (**a**) pillar-like DLIP structure with a period of 1.5 μm (DLIP-type 2). (**b**–**g**) Evolution of surface morphology on top of DLIP-type 2 structure with increasing peak fluence levels processed with the Trumpf TruMicro 5050 laser system.

**Figure 8:**
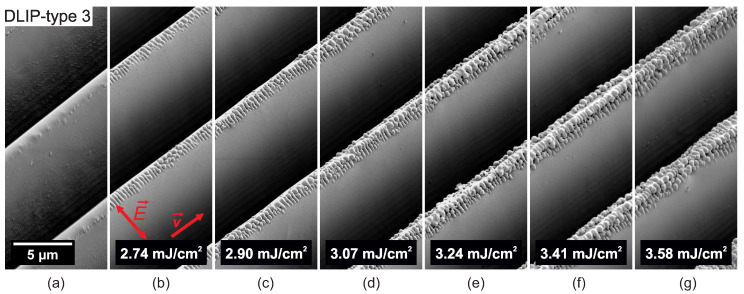
SEM micrographs of (**a**) ridge-like DLIP structure with a period of 10 μm (DLIP-type 3). (**b**–**g**) Evolution of surface morphology on top of the DLIP structure with increasing peak fluence levels and a laser polarization perpendicular to the DLIP ridges processed with the Trumpf TruMicro 5050 laser system.

**Figure 9:**
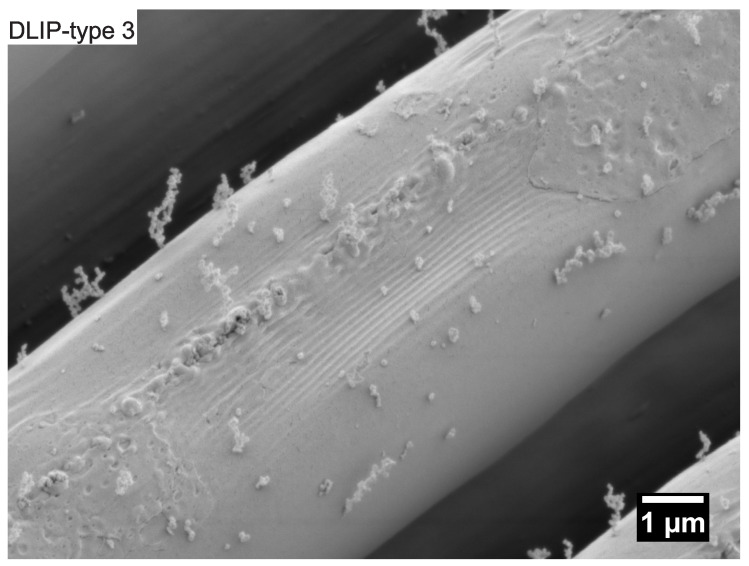
SEM micrograph of LSFL-II on top of the ridge of ridge-like DLIP structure with a period of 10 μm (DLIP-type 3) processed with the Time-Bandwidth Fuego laser system. This sample was contaminated.

**Figure 10:**
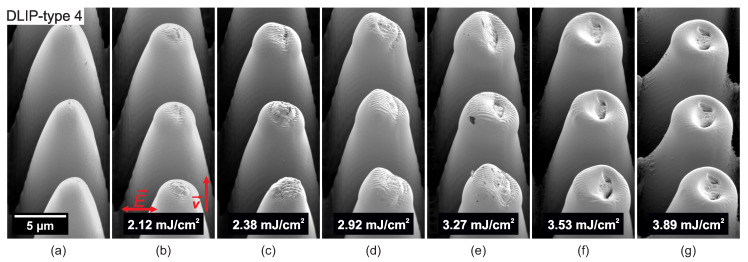
SEM micrographs of (**a**) pillar-like DLIP structure with a period of 10 μm (DLIP-type 4). (**b**–**g**) Evolution of surface morphology on top of the DLIP structure with increasing peak fluence levels processed with the Time-Bandwidth Fuego laser system.

In a further processing phase, the DLIP-treated PC samples were irradiated with ultrashort UV radiation with the aim to create a two-level hierarchical microtexture. Figure 5, Figure 6, Figure 7, Figure 8, Figure 9 and Figure 10 show SEM micrographs of different DLIP structures processed in a second laser processing step in order to produce LIPSS with orthogonal scanning (laser polarization) directions regarding the direction of the DLIP ridges and at various laser fluence levels, indicated as $\vec{E}$ and $\vec{v}$ in sub-figures (b), respectively.

It can be observed in Figure 5, that LSFL-II parallel to the laser polarization and perpendicular to the 1.5 μm DLIP ridges start to develop on the latter at a fluence level of 2.74 mJ/cm${}^{2}$ and higher. Also, nano-droplets start to appear in the valleys between the DLIP ridges (see Figure 5b). At a slightly higher laser fluence level of 3.07 mJ/cm${}^{2}$, LSFL-II also start to develop in the valleys between the DLIP ridges in form of periodic chains of nano-droplets (see Figure 5c). At a fluence of 3.24 mJ/cm${}^{2}$, all DLIP ridges are homogeneously covered with LSFL-II. Additionally, it can be observed that the nano-droplets between the DLIP ridges in Figure 5d–f are larger in diameter then at lower fluence levels in Figure 5b,c. The seeding of the periodic nano-droplet chains in Figure 5c can be linked to optical scattering effects, leading to periodic laser-induced defects of the PC with increased absorptivity [26]. The growth of the nano-droplets can be related to thermocapillary forces, pushing molten material from the ridges down in the DLIP valleys towards the nano-droplets [55,56]. At even higher laser fluence levels, nano-droplet appearance increases and the DLIP ridges become thinner due to more molten material that is transferred into nano-droplets until the ridges merge into each other and severe ablation takes place, see Figure 5e–g.

Figure 6 shows SEM micrographs of the evolution of surface morphology on top of the DLIP-type 1 structure with increasing peak fluence levels and a laser polarization parallel to the DLIP ridges. Here, the polarization of the laser beam is rotated by 90${}^{{}^{\circ}}$ compared to Figure 5, i.e., parallel to the DLIP ridges. It can be observed, that also in this case nano-droplets appear at the valleys of the DLIP ridges at a fluence level of 2.74 $\mathrm{mJ}/{\mathrm{cm}}^{2}$, see Figure 6b. At a somewhat higher fluence level of 2.90 $\mathrm{mJ}/{\mathrm{cm}}^{2}$, the DLIP ridges start to separate into chains of larger micro-droplets. Similar results were found when processing LSFL on top of LSFL on polyethersulfone, when the sample was rotated by 90${}^{{}^{\circ}}$ [57] and when processing LIPSS on a chromium thin film [58]. This phenomenon can be related to the Plateau-Rayleigh instability [58]. In brief, the surface energy in a stationary fluid in cylindrical form is larger than the effect of gravity and, hence, changes the shape of the cylinder into droplets in order to reduce the total surface energy. As for the case with the laser polarization perpendicular to the DLIP pattern (see Figure 5), the amount of nano-droplet increases with increasing laser fluence levels, see Figure 6d–g. LSFL-II appear inhomogeneously and randomly on top of DLIP ridges at fluence levels of 3.41 $\mathrm{mJ}/{\mathrm{cm}}^{2}$ and 3.58 $\mathrm{mJ}/{\mathrm{cm}}^{2}$. Similar results were found for the pillar-like DLIP structure with a period of 1.5 μm (DLIP-type 2), as shown in Figure 7. Here, nano-droplets start first to appear between the ridges and droplet appearance and growth dominates. LSFL-II were found sporadically on top of the droplet roughened surface at laser fluence levels of 2.57 to 3.07 $\mathrm{mJ}/{\mathrm{cm}}^{2}$, see Figure 7a–g.

The formation of LIPSS on top of polymeric photoresist film microstructures were recently reported by Ehrhardt et al. [42]. The authors studied the formation of LSFL-II on top of pillar-like dot array microstructures with pillar widths of 2 × 2 μm${}^{2}$ and 5 × 5 μm${}^{2}$ and a pillar height of about 2.2 μm, as well as on top of ridge-like microstructures with ridge widths of 1 μm and 3 μm and a ridge height of about 1 μm using 100 to 1500 pulses of a nanosecond laser source with a wavelength of 248 nm at a pulse repetition rate of 100 Hz. The authors reported that no laser parameter regime was found to obtain LSFL on top of 1 × 1 μm${}^{2}$ dot pillar arrays. This is in agreement with our experiments for the pillar-like DLIP-type 2 structure with a period of 1.5 μm reported in this study.

Figure 8 shows the evolution of nanostructures on top of ridge-like DLIP structures with a period of 10 μm (DLIP-type 3) when irradiated with UV picosecond pulses linearly polarized perpendicular to the ridges of the DLIP structure. At a laser fluence level of 2.74 mJ/cm${}^{2}$, LSFL-II and nano-droplets appear on top of the DLIP ridges, see Figure 8b. With increasing laser fluence, the nano-droplet growth on top of the ridges is reinforced, see Figure 8b–g. When irradiating the ridge-like DLIP structures having a period of 10 μm with the laser polarization parallel to the DLIP ridges, LSFL-II only appear on top of the DLIP ridges at laser fluence levels exceeding the melting threshold of the material. As a consequence, the “sharp” DLIP ridges collapse, forming small valleys as shown in Figure 9.

The formation of LSFL-II and deformation due to melting of pillar-like DLIP cones due to UV picosecond laser irradiation can be observed in Figure 10. It can be seen that the DLIP cones tip flattens due to laser-induced melting effects. Moreover, LSFL-II develop on top of the flattened parts, see Figure 10a–f. The melting also leads to a collapse of the DLIP cones and leaves some holes in the central region, see Figure 10e–g. The holes may be induced due to the sub-surface release of gaseous photo-thermal reaction products. Similar results were found in the recent publication by Ehrhardt et al. [42] on pre-patterned polymer films.

The appearance of LSFL-II on ridge-like photoresist film microstructures “moved” from the top to the side walls of the ridge-like structures with increasing laser fluence levels, as reported by Ehrhardt et al. [42]. Additionally, these authors reported that melting is responsible for the disappearance of the LSFL-II on top of the ridge-like microstructures [42]. The appearance of the LSFL-II only on top of the DLIP ridges and pillars in this study and ridge-like microstructures reported in [42] at certain fluence levels can both be explained with the decrease of the local laser fluence at the tilted slopes of the DLIP ridge topography. That is, due to the geometrical enlargement of the laser spot on the irradiated surface area at slopes for non-normal incident radiation, the laser fluence level decreases below the LSFL-II threshold.

**Figure 11:**
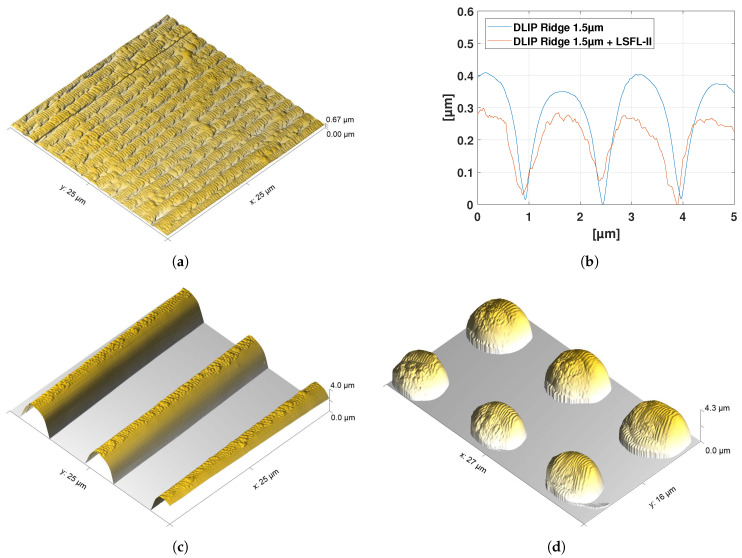
(**a**) AFM micrograph of LSFL-II processed on DLIP-type 1 with polarization perpendicular to the DLIP ridges at a laser peak fluence of $F_{0}=3.24\;\mathrm{mJ}/{\mathrm{cm}}^{2}$, (see Figure 5d). (**b**) AFM cross-sections of ridge-like DLIP structures with a period of 1.5 μm (DLIP-type 3)—with (see Figure 11a) and without LSFL-II. (**c**) AFM micrograph of LSFL-II processed on DLIP-type 3 with polarization perpendicular to the DLIP ridges at a laser peak fluence of $F_{0}=2.74\;\mathrm{mJ}/{\mathrm{cm}}^{2}$, (see Figure 8b). (**d**) AFM micrograph of LSFL-II processed on DLIP-type 4 at a laser peak fluence of $F_{0}=2.92\;\mathrm{mJ}/{\mathrm{cm}}^{2}$, (see Figure 10d).

In order to evaluate the periodicity and amplitude of the LSFL-II on top of the different DLIP structures, the topography of DLIP structures at which the ridges are covered homogeneously with LSFL-II are analyzed using AFM. Figure 11a,c,d show the AFM micrographs of Figure 5d, Figure 8b and Figure 10d, respectively. Note, that the DLIP-type 3 and 4 are too deep for the AFM tip to reach the bottom of the DLIP ridges. For these two cases, the AFM micrograph is cut at the depth at which the AFM measurement lost its signal. The average periods of the LSFL-II are $\Lambda_{\mathrm{LIPSS}}=254\;\pm \;$9 nm and the average amplitudes are 33 ± 12 nm for all analyzed hierarchical structures. It is known that LSFL-II are seeded and formed in a sub-surface layer [26,27]. Figure 11b shows cross-sections obtained from AFM measurements of ridge-like DLIP structures with a period of 1.5 μm (DLIP-type 3)—with and without LSFL-II, as it can be seen in Figure 11a. The Figure also shows, that the overall depth of the of the DLIP structure is reduced when LSFL-II are generated. This is an indication, that the LSFL-II indeed are formed below the surface.

### 3.4. FDTD Simulations

**Figure 12:**
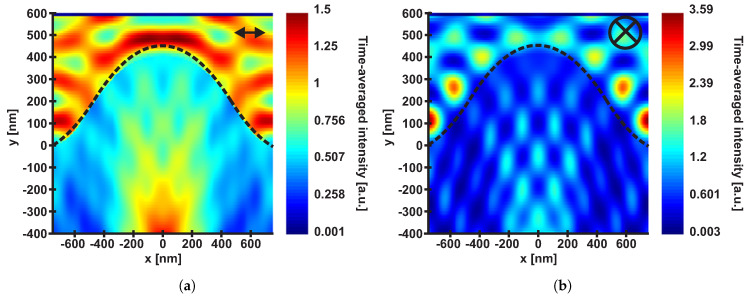
Calculated time-averaged optical intensity distribution of a 6.7 ps laser pulse with a wavelength of 343 nm with a polarization (**a**) perpendicular and (**b**) parallel to the orientation of DLIP-type 1 ridges with a period of 1.5 μm. The arrows in the upper right corners indicate the direction of the laser polarization. Note the different color scales used in (**a**,**b**) for encoding the intensity.

Figure 12 shows the time-averaged optical intensity distribution (as calculated by the photonic simulation software, see Section 2.4) induced by one UV picosecond pulse on and in the surface of DLIP-type 1 structure with the parameters described in Section 2.4. It can be concluded from this Figure, that the maximum intensities differ for each case of polarization. That is, if the orientation of the laser polarization is perpendicular to the DLIP ridges, the maximum intensity is found on top of the ridge and another less localized intensity enhancement is observed several hundreds of nanometers below it. The intensity maximum close to the surface can facilitate the seeding of LIPSS on top of the DLIP ridges, see Figure 5b. If the orientation of the laser polarization is parallel to the DLIP ridges, the maximum intensity is found in the bottom of the DLIP ridges, which can explain the dominance of nano-droplet growth at these positions, see Figure 6b.

### 3.5. Chemical Characterization

**Figure 13:**
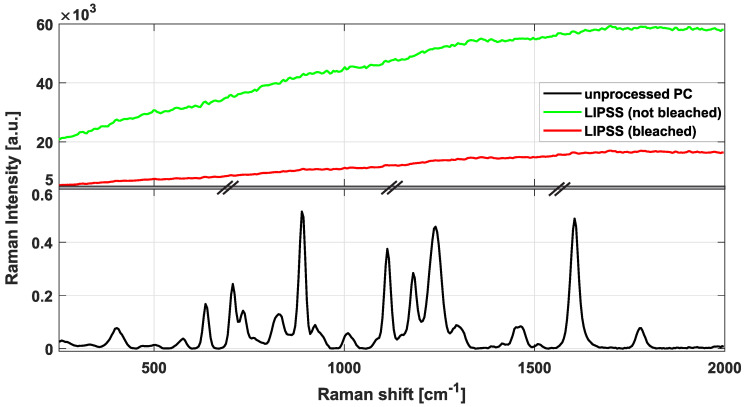
Raman spectra of non-irradiated (unprocessed) PC (black curve) and of UV ps-laser irradiated PC with LIPSS (LSFL-II) as described in Section 3.2 before (green curve) and after (red curve) photo-bleaching (for details see the text). Note that the ordinate is separated and the scaling differs for both separations.

Figure 13 shows the μ-Raman spectra of non-irradiated and LIPSS-processed PC (LSFL-II as described in Section 3.2). The reference measurement of the non-irradiated material (black curve) shows pronounced characteristic peaks that are typical for this type of bisphenol-A based PC material [59]. The measurement in the LIPSS-covered area (green and red curves) exhibit a very strong and broad background signal that is caused by optical fluorescence. This fluorescence is excited by the Raman laser in the laser-modified PC over the entire depth (Rayleigh-length, ⪆3 μm for the given microscope objective) of the probing Raman spot. Moreover, it was noticed that the μ-RS spectra recorded in the LIPSS-covered areas show a characteristic photo-bleaching effect, i.e., the fluorescence level of the spectra drops about 75 percent upon exposure to the Raman laser radiation and then saturates after several tens to hundreds of seconds, see Figure 13. The effect arises from broken bonds in the polymer material that create energetic states within the electronic band gap. These states are capable of being excited by the Raman laser radiation at 532 nm wavelength, causing the strong fluorescence background. Upon continuous Raman laser irradiation, these broken bonds may react with the environment (e.g., via oxidation), hence, reducing the fluorescence again. The red curve of the laser-processed PC was recorded after photo-bleaching the sample. However, at the given signal-to-noise level it is difficult to quantify changes induced upon the UV ps-laser irradiation here. Hence, other DLIP structures were not tested by μ-RS here and a more surface sensitive method (ATR-FTIR) was selected for further material characterizations.

**Figure 14:**
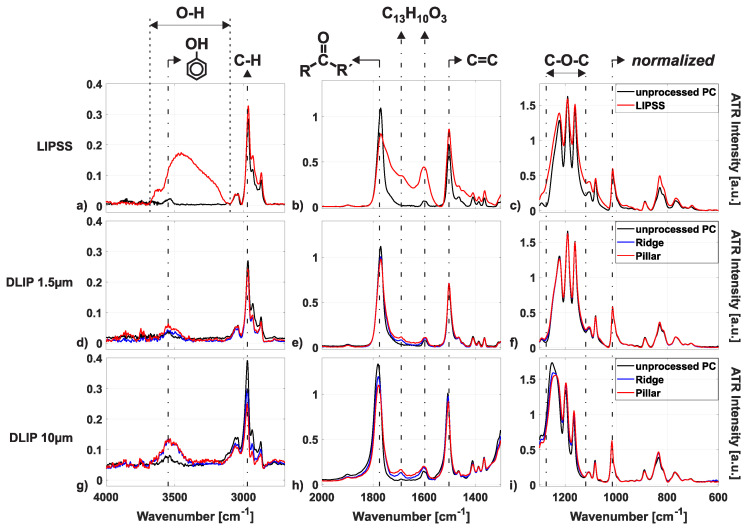
ATR-FTIR spectra of unprocessed samples (black curves) compared to samples processed homogeneously with LIPSS (type LSFL-II, red curves in (**a**–**c**), ridge-like (blue curves) or pillar-like (red curves) DLIP structures with 1.5 μm period (**d**–**f**) and ridge-like (blue curves) or pillar-like (red curves) DLIP structures with 10 μm period (**g**–**i**). Note the different vertical scales in the graphs.

Fourier-transform infrared spectroscopy in attenuated total reflection mode (ATR-FTIR) is capable of selectively examining the near-surface layer of organic films [46]. With this technique, the chemical changes before and after laser irradiation and the resulting degradation of the polycarbonate are analyzed. Figure 14 exemplifies ATR-FTIR spectra of non-irradiated polycarbonate samples (black curves) compared to samples processed homogeneously with LSFL-II (see Figure 14a–c, top row, red curves), DLIP structures with a period of 1.5 μm (see Figure 14d–f, middle row, red and blue curves) and DLIP structures with a period of 10 μm (see Figure 14g–i, bottom row, red and blue curves). For each type of surface structure, three different spectral regions of interest are selected, i.e., left column: 4000 to 2700 cm${}^{-1}$, middle column: 2000 to 1300 cm${}^{-1}$, and right column: 1300 to 600 cm${}^{-1}$, all being representative for the absorption range of specific vibrational excitation modes in the polymeric material. All measured ATR-FTIR spectra were normalized at the peak located at 1014 cm${}^{-1}$, as proposed in Ref. [46]. It can be concluded from the graphs that the different laser processing techniques and irradiation parameters lead to a degradation of characteristic absorption bands and the formation of various absorption bands at specific wavenumbers. The differences of the reference spectra of the unprocessed polycarbonate among the different measurements may arise from inhomogeneities and additives within the polycarbonate samples.

For all processed samples, the laser processing led to a degradation of the C-H vibrational region at a wavenumber of about 3000 cm${}^{-1}$ (see Figure 14a,d,f), the carbonyl and C=C vibration peaks at wavenumbers about 1790 and 1500 cm${}^{-1}$, respectively (see Figure 14b,e,h) and the C-O-C vibration features at about 1120 to 1280 cm${}^{-1}$ (see Figure 14c,f,i). The degradation of the laser irradiated polycarbonate is also accompanied by the appearance/growth of other bands. While the processing of LIPSS leads to a broad absorption band at about 3400 cm${}^{-1}$ (see Figure 14a), which is related to the OH-stretch region [45], the processing of DLIP structures leads to absorption bands at 3550 and 3500 cm${}^{-1}$ (see Figure 14d,g), which are attributed to free- and hydrogen-bonded phenolic groups [44]. Additionally, two bands arise at about 1630 and 1690 cm${}^{-1}$ (see Figure 14b,e,h), which are attributed to the creation of phenylsalicate and dihydroxybenzophenone (both $C_{13}H_{10}O_{3}$), respectively [46,47]. The degradation on the polycarbonate upon irradiation with ultrashort pulsed UV radiation is in full accordance with the literature here [44,45,46,47].

It can be observed in Figure 14, when comparing the relative changes of the absorption spectra from the untreated PC with the spectra of the processed samples with various micro- and nanostructures, that the relative changes due to laser irradiation are most affected by the production of homogeneous areas of LIPSS (see Figure 14a–c) and least affected by the creation of DLIP structures with a period of 1.5 μm (see Figure 14d–f). Additionally, it can be observed in Figure 14d–i, that the formation of pillar-like DLIP structures affects the degradation of the PC more than the fabrication of ridge-like DLIP structures. The different levels of degradation of the PC due to the processing of different structures can be related to the number of laser pulses irradiating one spot diameter $N_{\mathrm{eff}}$ and the corresponding accumulated fluence ($F_{\mathrm{acc}}$) levels, see Table 2. The total amount of pulses impinging the same spot equals $N_{\mathrm{eff}}=N_{\mathrm{OS}}\times d^{2}\times f/\left(v\times h\right)$ with *h* being the hatch distance (line pitch). The accumulated fluence is given by $F_{\mathrm{acc}}=N_{\mathrm{eff}}\times F_{0}$. It can be observed in and Table 2, that although the peak fluence to manufacture LIPSS-II is much less than the peak fluence to create the ridge-like and pillar-like DLIP structures, the accumulated fluence is much greater when comparing those parameters. The latter explains the increasing degradation of the polycarbonate when comparing different ATR-FTIR spectra of the different samples in Figure 14.

It must to be noted, that the light penetration depth according to the Lambert-Beer law between the nanosecond and picosecond laser sources differ due to the different wavelength used. The penetration depth will also have an impact on the depth of the changes of the chemical structure of the polymer. Whereas the penetration depth for a wavelength of 266 nm on PC is about 0.6 μm [60], the penetration depths for wavelengths of 343 nm and 355 nm are about 104 and 107 μm, respectively [54]. However, the depth of the chemical changes fall out of the scope of this paper.

## 4. Conclusions

The evolution of nanometric LIPSS on different types of micrometric DLIP structures with increasing fluence levels was analyzed to achieve hierarchical micro-/nano-structures on a commercial polycarbonate (Makrolon ™). It was found that LIPSS can be formed on top of various forms and sizes of DLIP structures by selecting the laser beam polarization perpendicular to the DLIP ridges. However, the fabrication of LIPSS on a micro-scale structure is limited by the height and width of the pre-processed microscale ridges. FDTD calculations of the time-averaged optical intensity distribution of a picosecond laser pulse with a wavelength of 343 nm at micro-structures with a period of 1.5 μm and a height of 400 nm were conducted with varying laser beam polarization directions. If the latter was set perpendicular to the micro-structures, the time-averaged optical intensity was found to be enhanced on top of the micro-structures, promoting the seeding of LSFL on top of the ridges. However, when the laser beam polarization direction was set parallel to the DLIP ridges, the optical intensity was found to be locally increased at the bottom of the DLIP ridges, enhancing nano-droplet growth at these positions. Moreover, since LSFL appearance is limited to a narrow window of laser fluence levels, the growth of LSFL only on top of the DLIP ridges was was limited by the non-normal angle of incidence of the laser radiation at the side walls of the DLIP structures. The latter decreases the local fluence level below the LSFL threshold. As an important aspect for potential future applications, it was found that a sufficiently broad top of the DLIP-pillars is required to allow LIPSS to be formed there. Additionally, with increasingly accumulated fluence levels, the degradation of the polymer also progresses. This needs to be considered if the original property of the unprocessed polymer is essential for applications.

## Figures and Tables

**Table 1:** Laser sources and process parameters to produce hierarchical structures.

Structure Type	DLIP		LIPSS	
Laser source	Laser-export *TECH-263 Advanced*		Trumpf *TruMicro 5050*	Time-Bandwidth *Fuego*
DLIP Period [µm]	1.5	10	-	-
Wavelength [nm]	266	266	343	355
Pulse Duration [s]	$3\times 10^{-9}$	$3\times 10^{-9}$	$7\times 10^{-12}$	$1\times 10^{-11}$
Pulse Frequency [kHz]	2	2	100	100
Beam Quality Factor M${}^{2}$ [-]	<1.3	<1.3	<1.3	<1.3
Laser Spot Diameter [µm]	25	25	174	120
Scan Velocity [mm/s]	2.5	2.5	1000	600
Number of Overscans [-]	1	1	1000	1000
Line Pitch [µm]	7.33	10.16	10	3
Geometrical Pulse-to-Pulse Overlap [%]	85	90	94	95
Peak Fluence Levels [J/cm${}^{2}$]	0.25 (ridges) + 0.2 (Pillars)	1.45 (ridges) + 1.13 (Pillars)	2…4$\times 10^{-3}$	2…4$\times 10^{-3}$

**Table 2:** Effective number of pulses and accumulated laser fluence levels due to various laser micro- and nanostructuring techniques.

Structure Type	$N_{\mathrm{eff}}$ [-]	$F_{0}\;\left[J/{\mathrm{cm}}^{2}\right]$	$F_{\mathrm{acc}}\;\left[J/{\mathrm{cm}}^{2}\right]$
LSFL-II	302760	4.4$\times 10^{-3}$	1338
DLIP 1.5 μm Ridge	68	0.25	17
DLIP 1.5 μm Pillar	68 + 68	0.25 + 0.2	27
DLIP 10 μm Ridge	50	1.45	71
DLIP 10 μm Pillar	50 + 50	1.45 + 1.13	111

## References

[1] Barthlott W., Neinhuis C. (1997). Purity of the sacred lotus, or escape from contamination in biological surfaces. Planta.

[2] Hasan J., Webb H.K., Truong V.K., Pogodin S., Baulin V.A., Watson G.S., Watson J.A., Crawford R.J., Ivanova E.P. (2013). Selective bactericidal activity of nanopatterned superhydrophobic cicada Psaltoda claripennis wing surfaces. Appl. Microbiol. Biotechnol..

[3] Pogodin S., Hasan J., Baulin V.A., Webb H.K., Truong V.K., Phong Nguyen T.H., Boshkovikj V., Fluke C.J., Watson G.S., Watson J.A. (2013). Biophysical model of bacterial cell interactions with nanopatterned cicada wing surfaces. Biophys. J..

[4] Bandara C.D., Singh S., Afara I.O., Wolff A., Tesfamichael T., Ostrikov K., Oloyede A. (2017). Bactericidal effects of natural nanotopography of Dragonfly Wing on Escherichia coli. ACS Appl. Mater. Interfaces.

[5] Jaggessar A., Shahali H., Mathew A., Yarlagadda P.K. (2017). Bio-mimicking nano and micro-structured surface fabrication for antibacterial properties in medical implants. J. Nanobiotechnology.

[6] Parker A.R., Lawrence C.R. (2001). Water capture by a desert beetle. Nature.

[7] Hermens U., Kirner S.V., Emonts C., Comanns P., Skoulas E., Mimidis A., Mescheder H., Winands K., Krüger J., Stratakis E. (2017). Mimicking lizard-like surface structures upon ultrashort laser pulse irradiation of inorganic materials. Appl. Surf. Sci..

[8] Lutey A.H., Gemini L., Romoli L., Lazzini G., Fuso F., Faucon M., Kling R. (2018). Towards laser-textured antibacterial surfaces. Sci. Rep..

[9] van der Poel S., Mezera M., Römer G.R.B.E., de Vries E., Matthews D. (2019). Fabricating Laser-Induced Periodic Surface Structures on Medical Grade Cobalt–Chrome–Molybdenum: Tribological, Wetting and Leaching Properties. Lubricants.

[10] Qin L., Wu H., Guo J., Feng X., Dong G., Shao J., Zeng Q., Zhang Y., Qin Y. (2018). Fabricating hierarchical micro and nano structures on implantable Co–Cr–Mo alloy for tissue engineering by one-step laser ablation. Colloids Surfaces B Biointerfaces.

[11] Heitz J., Plamadeala C., Muck M., Armbruster O., Baumgartner W., Weth A., Steinwender C., Blessberger H., Kellermair J., Kirner S.V. (2017). Femtosecond laser-induced microstructures on Ti substrates for reduced cell adhesion. Appl. Phys. A Mater. Sci. Process..

[12] Paradisanos I., Fotakis C., Anastasiadis S.H., Stratakis E. (2015). Gradient induced liquid motion on laser structured black Si surfaces. Appl. Phys. Lett..

[13] Stark T., Kiedrowski T., Marschall H., Lasagni A.F. (2019). Avoiding starvation in tribocontact through active lubricant transport in laser textured surfaces. Lubricants.

[14] Eichstädt J., Römer G.R.B.E., Huis in’t Veld A.J. (2011). Towards friction control using laser-induced periodic Surface Structures. Phys. Procedia.

[15] Bonse J., Kirner S.V., Griepentrog M., Spaltmann D., Krüger J. (2018). Femtosecond laser texturing of surfaces for tribological applications. Materials.

[16] Alamri S., Aguilar-Morales A.I., Lasagni A.F. (2018). Controlling the wettability of polycarbonate substrates by producing hierarchical structures using Direct Laser Interference Patterning. Eur. Polym. J..

[17] Aguilar-Morales A.I., Alamri S., Lasagni A.F. (2018). Micro-fabrication of high aspect ratio periodic structures on stainless steel by picosecond direct laser interference patterning. J. Mater. Process. Technol..

[18] Vorobyev A.Y., Guo C. (2008). Colorizing metals with femtosecond laser pulses. Appl. Phys. Lett..

[19] Dusser B., Sagan Z., Soder H., Faure N., Colombier J., Jourlin M., Audouard E. (2010). Controlled nanostructures formation by ultra fast laser pulses for color marking. Opt. Express.

[20] Ahsan M.S., Ahmed F., Kim Y.G., Lee M.S., Jun M.B.G. (2011). Colorizing stainless steel surface by femtosecond laser induced micro/nano-structures. Appl. Surf. Sci..

[21] Ionin A.A., Kudryashov S.I., Makarov S.V., Seleznev L.V., Sinitsyn D.V., Golosov E.V., Golosova O.A., Kolobov Y.R., Ligachev A.E. (2012). Femtosecond laser color marking of metal and semiconductor surfaces. Appl. Phys. A Mater. Sci. Process..

[22] Alamri S., Fraggelakis F., Kunze T., Krupop B., Mincuzzi G., Kling R., Lasagni A.F. (2019). On the interplay of DLIP and LIPSS upon ultra-short laser pulse irradiation. Materials.

[23] Fabris D., Lasagni A.F., Fredel M.C., Henriques B. (2019). Direct Laser Interference Patterning of Bioceramics: A Short Review. Ceramics.

[24] Lang V., Roch T., Lasagni A.F. (2016). High-Speed Surface Structuring of Polycarbonate Using Direct Laser Interference Patterning: Toward 1 m^2^ min^−1^ Fabrication Speed Barrier. Adv. Eng. Mater..

[25] Bonse J., Höhm S., Kirner S.V., Rosenfeld A., Krüger J. (2017). Laser-induced periodic surface structures—A scientific evergreen. IEEE J. Sel. Top. Quantum Electron..

[26] Rudenko A., Colombier J.P., Höhm S., Rosenfeld A., Krüger J., Bonse J., Itina T.E. (2017). Spontaneous periodic ordering on the surface and in the bulk of dielectrics irradiated by ultrafast laser: A shared electromagnetic origin. Sci. Rep..

[27] Mezera M., Bonse J., Römer G.R.B.E. (2019). Influence of bulk temperature on laser-induced periodic surface structures on polycarbonate. Polymers.

[28] Mezera M., Römer G.R.B.E. (2019). Model based optimization of process parameters to produce large homogeneous areas of laser-induced periodic surface structures. Opt. Express.

[29] Kirner S.V., Hermens U., Mimidis A., Skoulas E., Florian C., Hischen F., Plamadeala C., Baumgartner W., Winands K., Mescheder H. (2017). Mimicking bug-like surface structures and their fluid transport produced by ultrashort laser pulse irradiation of steel. Appl. Phys. A Mater. Sci. Process..

[30] Dufft D., Rosenfeld A., Das S.K., Grunwald R., Bonse J. (2009). Femtosecond laser-induced periodic surface structures revisited: A comparative study on ZnO. J. Appl. Phys..

[31] Bonse J., Sturm H., Schmidt D., Kautek W. (2000). Chemical, morphological and accumulation phenomena in ultrashort-pulse laser ablation of TiN in air. Appl. Phys. A Mater. Sci. Process..

[32] Yasumaru N., Miyazaki K., Kiuchi J. (2003). Femtosecond-laser-induced nanostructure formed on hard thin films of TiN and DLC. Appl. Phys. A Mater. Sci. Process..

[33] Florian C., Déziel J.L., Kirner S.V., Siegel J., Bonse J. (2020). The role of the laser-induced oxide layer in the formation of laser-induced periodic surface structures. Nanomaterials.

[34] Baudach S., Bonse J., Kautek W. (1999). Ablation experiments on polyimide with femtosecond laser pulses. Appl. Phys. A: Mater. Sci. Process..

[35] Rebollar E., Pérez S., Hernández J.J., Martín-Fabiani I., Rueda D.R., Ezquerra T.A., Castillejo M. (2011). Assessment and formation mechanism of laser-induced periodic surface structures on polymer spin-coated films in real and reciprocal space. Langmuir.

[36] Castillejo M., Ezquerra T.A., Martín M., Oujja M., Pérez S., Rebollar E. Laser nanostructuring of polymers: Ripples and applications. Proceedings of the AIP Conference Proceedings; American Institute of Physics (AIP).

[37] Mezera M., van Drongelen M., Römer G.R.B.E. (2018). Laser-Induced Periodic Surface Structures (LIPSS) on polymers processed with picosecond laser pulses. J. Laser Micro Nanoeng..

[38] Ionin A.A., Kudryashov S.I., Makarov S.V., Rudenko A.A., Seleznev L.V., Sinitsyn D.V., Golosov E.V., Kolobov Y.R., Ligachev A.E. (2013). Beam spatial profile effect on femtosecond laser surface structuring of titanium in scanning regime. Appl. Surf. Sci..

[39] Kunz C., Büttner T.N., Naumann B., Boehm A.V., Gnecco E., Bonse J., Neumann C., Turchanin A., Müller F.A., Gräf S. (2018). Large-area fabrication of low- and high-spatial-frequency laser-induced periodic surface structures on carbon fibers. Carbon.

[40] Vorobyev A.Y., Guo C. (2013). Direct femtosecond laser surface nano/microstructuring and its applications. Laser Photonics Rev..

[41] Klein-Wiele J.H., Blumenstein A., Simon P., Ihlemann J. (2020). Laser interference ablation by ultrashort UV laser pulses via diffractive beam management. Adv. Opt. Technol..

[42] Ehrhardt M., Lai S., Lorenz P., Zimmer K. (2020). Guiding of LIPSS formation by excimer laser irradiation of pre-patterned polymer films for tailored hierarchical structures. Appl. Surf. Sci..

[43] Abts G., Eckel T., Wehrmann R. (2014). Polycarbonates.

[44] Rivaton A., Sallet D., Lemaire J. (1986). The photo-chemistry of bisphenol-A polycarbonate reconsidered: Part 2—FTIR Analysis of the Solid-state Photo-chemistry in ‘Dry’ Conditions. Polym. Degrad. Stab..

[45] Adams M.R., Garton A. (1993). Surface modification of bisphenol-A-polycarbonate by far-UV radiation. Part I: In vacuum. Polym. Degrad. Stab..

[46] Diepens M., Gijsman P. (2007). Photodegradation of bisphenol A polycarbonate. Polym. Degrad. Stab..

[47] Yazdan Mehr M., Van Driel W.D., Jansen K.M., Deeben P., Boutelje M., Zhang G.Q. (2013). Photodegradation of bisphenol A polycarbonate under blue light radiation and its effect on optical properties. Opt. Mater..

[48] Liu J.M. (1982). Simple technique for measurements of pulsed Gaussian-beam spot sizes. Opt. Lett..

[49] Dyer P.E., Jenkins S.D., Sidhu J. (1986). Development and origin of conical structures on XeCl laser ablated polyimide. Appl. Phys. Lett..

[50] Lippert T., Dickinson J.T. (2003). Chemical and spectroscopic aspects of polymer ablation: Special features and novel directions. Chem. Rev..

[51] Murthy N.S., Prabhu R.D., Martin J.J., Zhou L., Headrick R.L. (2006). Self-assembled and etched cones on laser ablated polymer surfaces. J. Appl. Phys..

[52] Mezera M., Römer G.R.B.E. (2019). Upscaling laser-induced periodic surface structures (LIPSS) manufacturing by defocused laser processing. SPIE Conference Proceedings.

[53] The MathWorks, Inc. (2019). MATLAB^®^ R2019b.

[54] Brissinger D. (2019). Complex refractive index of polycarbonate over the UV-Vis-IR region from 0.2 to 3 μm. Appl. Opt..

[55] Kuchmizhak A.A., Vitrik O.B., Kulchin Y.N. (2014). Novel hydrodynamic instability of the molten Au/Pd alloy film irradiated by tightly focused femtosecond laser pulses. Pac. Sci. Rev..

[56] Wang H.P., Guan Y.C., Zheng H.Y., Hong M.H. (2019). Controllable fabrication of metallic micro/nano hybrid structuring surface for antireflection by picosecond laser direct writing. Appl. Surf. Sci..

[57] Fajstavr D., Michaljaničová I., Slepička P., Neděla O., Sajdl P., Kolská Z., Švorčík V. (2018). Surface instability on polyethersulfone induced by dual laser treatment for husk nanostructure construction. React. Funct. Polym..

[58] Gedvilas M., Račiukaitis G., Kučikas V., Regelskis K. (2012). Driving forces for self-organization in thin metal films during their partial ablation with a cylindrically focused laser beam. AIP Conference Proceedings.

[59] Dybal J., Schmidt P., Baldrian J., Kratochvíl J. (1998). Ordered structures in polycarbonate studied by infrared and Raman spectroscopy, wide-angle X-ray scattering, and differential scanning calorimetry. Macromolecules.

[60] Philipp H.R., Legrand D.G., Cole H.S., Liu Y.S. (1987). The Optical Properties of Bisphenol-A Polycarbonate. Polym. Eng. Sci..

